# Investigation of Snoring and Obstructive Sleep Apnea Using Portable Polysomnography in Patients with Temporomandibular Disorder

**DOI:** 10.26502/droh.0050

**Published:** 2022-09-30

**Authors:** Yeon-Hee Lee, Q-Schick Auh, Eun-Jae Chung

**Affiliations:** 1 Department of Orofacial Pain and Oral Medicine, Kyung Hee University Dental Hospital, Kyung Hee Medical center, Kyung Hee University, Seoul, Korea; 2 Otorhinolaryngology-Head & Neck Surgery, SNUCM Otorhinolaryngology-Head & Neck Surgery, Seoul National University Hospital Otorhinolaryngology-Head & Neck Surgery, Seoul, Korea

**Keywords:** Snoring, Obstructive sleep apnea, Mallampati score, Overweight, Polysomnography, Temporomandibular disorder

## Abstract

**Objective:**

To investigate snoring and obstructive sleep apnea (OSA) in patients with temporomandibular disorder (TMD) using portable polysomnography and identify sex-based differences in clinical features and sleep-related results

**Methods:**

Seventy consecutive patients (44 female; mean age, 46.6918.18 years) with myofascial pain-associated TMD, diagnosed based on the criteria for TMD Axis I, were enrolled. Sleep quality and quantity were measured using portable polysomnography. Clinical characteristics were investigated using well-structured standardized reports on clinical signs and symptoms, questionnaires, and clinical examination by TMD specialists.

**Results:**

Among 70 TMD patients, 50.0% had OSA and 15.7% had snoring, with no sex-based differences. The mean Mallampati scores for OSA prediction (2.69±1.12 vs. 1.70±0.82, p<0.001), mean body mass index (BMI) (24.94±1.78 vs. 22.02±2.24, p<0.001), and ratio of overweight patients (57.7 vs. 11.4%) with BMI ≥25 were significantly higher in males than in females (all p<0.001). Conversely, the mixed sleep apnea index was significantly higher in females than in males (0.81±0.80 vs. 0.44±0.54, p=0.022). Female sex was associated with the absence of snoring (OR=0.146, p=0.022). Based on the area under curve (AUC) value for snoring prediction, Mallampati score was the strongest predictor (AUC>0.932, p<0.001), followed by BMI, overweight, and obstructive sleep apnea index (AUC>0.8, all p<0.001).

**Conclusions:**

Our results support the necessity of investigating sex-based differences when examining sleep problems, including snoring and OSA, in TMD patients. Mallampati scoring could be a useful tool for physical examination prior to polysomnography. Sleep and biopsychosocial factors are important for the diagnosis and treatment of TMD.

## Introduction

Temporomandibular disorder (TMD) is a collective term for conditions characterized by pain and dysfunction of the temporomandibular joint (TMJ), masticatory muscles, and the surrounding structures. Symptoms of TMD include TMJ pain, masticatory muscle pain, TMJ noise, mouth-opening limitation, headache, sleep problems, and accompanying psychological deterioration [1]. The prevalence of TMD varies widely with the study methodology, ranging between 5% and 87% [2,3]. Female patients have TMD signs and symptoms more frequently than male patients, and the male-to-female ratio is approximately 1:2 [4]. With a complex etiology, TMD is not easily identified and resolved and hence becomes chronic [5]. To successfully diagnose and treat patients with TMD, they must be comprehensively identified in their respective biopsychosocial models. Sex is a crucial factor for pain; hormonal system, immunity, psychological axis, bodily changes with age, and pain control mechanisms can differ with it [6]. Thus, patients can have different signs and symptoms depending on their sex.

Ninety percent of patients with TMD experience poor sleep quality [7]. In 2014, the Diagnostic Criteria for TMD (DC/TMD) was published as a diagnostic tool for 12 common TMDs, including arthrogenous and myogenous TMD, and TMD attributed to TMD [8]. Currently, DC/TMD is the most widely used diagnostic criterion for TMD in clinical and research fields worldwide. DC/TMD is composed of dual axes: Axis I deals with the physical part, and Axis II deals with the psychosocial part, without consideration for sleep. Although sleep deterioration is supposedly substantial in TMD patients [9], the type and magnitude of sleep problems are not clearly known because of the numerous methodological limitations. Laboratory-based polysomnography (PSG) is considered the gold standard for objectively measuring sleep but is impractical for home utilization [10]. Portable PSG devices are an alternative to laboratory-based PSG, allowing objective sleep measurements in a home environment. In this study, we investigated sleep and its sex-based differences in patients with TMD using a portable PSG.

Obstructive sleep apnea (OSA) is a representative sleep disorder that involves a significant decrease or cessation of airflow in the presence of breathing effort. OSA is an upper airway dysfunction during sleep characterized by increased respiratory effort secondary to snoring and/or greater upper airway resistance and pharyngeal collapse [11]. At an apnea-hypopnea index (AHI) of ≥5 events/h, the prevalence ranges from 9% to 38% in the general population [12]. OSA affects both men (15%) and women (5%), and its prevalence is higher in men [13]. Sex-based differences exist in OSA signs and symptoms; male sex, a higher body mass index (BMI) and waist-to-hip ratio, greater neck circumference, arterial hypertension, older age, smoking, and snoring are closely related to OSA [14]. The common clinical complaints of OSA patients and their sleep partners are daytime sleepiness, snoring, gasping, and choking [15]. In the pathophysiological mechanism, there may be a marked decrease (hypopnea) or absence (apnea) of airflow in the nose and/or mouth, which is usually accompanied by oxyhemoglobin desaturation, and is usually terminated by a brief micro-arousal. Recurrent episodes of apnea lead to a decrease in slow-wave sleep and rapid eye movement (REM) sleep, ultimately leading to sleep disturbances and fragmentation [16]. OSA is a risk and/or persistence factor for chronic pain including TMD [17,18]. However, few studies have addressed snoring, a common symptom of OSA, in patients with TMD. Measuring periodic snoring at home is a simple and useful method for predicting OSA [19].

The first goal of this study was to objectively investigate sleep in patients with TMD using portable PSG. TMD and OSA are also reported to be comorbid conditions. The purpose of this study was to conduct an in-depth investigation of snoring, which may be the representative and early symptom of OSA, in patients with TMD. We hypothesized that the signs and symptoms of both diseases and the increase in BMI or accompanying collapsibility of the upper airway (obstructiveness) may differ depending on sex and have varying relationships with TMD. Finally, we intended to examine the relationship between sleep-related parameters and their clinical characteristics in TMD patients and investigate whether there are sex-based differences.

## Materials and Methods

### Participants

The research protocol was reviewed in accordance with the Declaration of Helsinki and approved by the Institutional Review Board of Kyung Hee University Dental Hospital (KHD IRB no.1804–2). Written informed consent was obtained from all participants. Informed consent was obtained to publish de-identified images of one participant who performed portable PSG in an online, open-access publication.

To investigate this research purpose, we conducted an observational study in clinical practice. A total of 70 consecutive patients (44 female; mean age, 46.69±18.18 years) with myofascial pain-associated TMD were included. We identified patients with TMD who visited the Department of Orofacial Pain and Oral Medicine at Kyung Hee University Dental Hospital of Seoul from October 1, 2018, to May 31, 2022. The patients were diagnosed with TMD using DC/TMD for TMD Axis I [8].

The inclusion criteria for patients with TMD were as follows: completed a set of routine TMJ assessments, performed portable PSG, completed sleep reports and constructive questionnaires, and no other treatment of the current episode, except medication. The exclusion criteria were as follows: serious injuries such as facial fractures and unstable multiple traumas, previous injury, neurological disorder unrelated to the trauma, musculoskeletal disorder before the injury, rheumatism, psychological problems, and pregnancy.

To assess the impact of sex on the distribution of demographics, clinical factors for signs and symptoms of TMD, and the presence of snoring and OSA, all variables were compared between the male and female TMD groups.

### TMD classification and clinical evaluation

Clinical evaluation procedures included an oral examination, interview, panoramic radiography, and a comprehensive questionnaire on DC/TMD Axis I diagnostic algorithms for TMD. Myofascial pain was diagnosed based on the DC/TMD Axis I algorithm.

### Characteristics of pain

Patients reported the duration of symptoms related to the masticatory muscles and adjacent structures based on the number of days elapsed since the first experience of symptoms related to TMD. TMD-related pain was scored subjectively by patients, ranging from 0 (no pain at all) to 10 (worst pain imaginable), using the visual analog scale (VAS).

### Contributing factors for TMD

We investigated self-reported parafunctional activities, including bruxism, using the Oral Behavior Checklist [20]. Self-assessment of sleep problems, headaches, psychological distress, and tinnitus have also been reported. Self-reported macrotrauma experience was evaluated using the dichotomous question, “Do you have any macrotrauma experience associated with current TMD?” Each parameter was recorded as a binary answer (yes or no) for all patients.

### Body mass index

Self-reported weight and height were used to assess overweight and obesity, based on the body mass index (BMI). To obtain BMI, the patient’s weight (kg) was divided by the square of the height (m). Major adult BMI categories are underweight (<18.5 kg/m^2^), normal weight (18.5–24.9), overweight (25–29.9), and obese (≥30) [21]. None of the participants in this study were obese.

### Mallampati score

**Figure 1:**
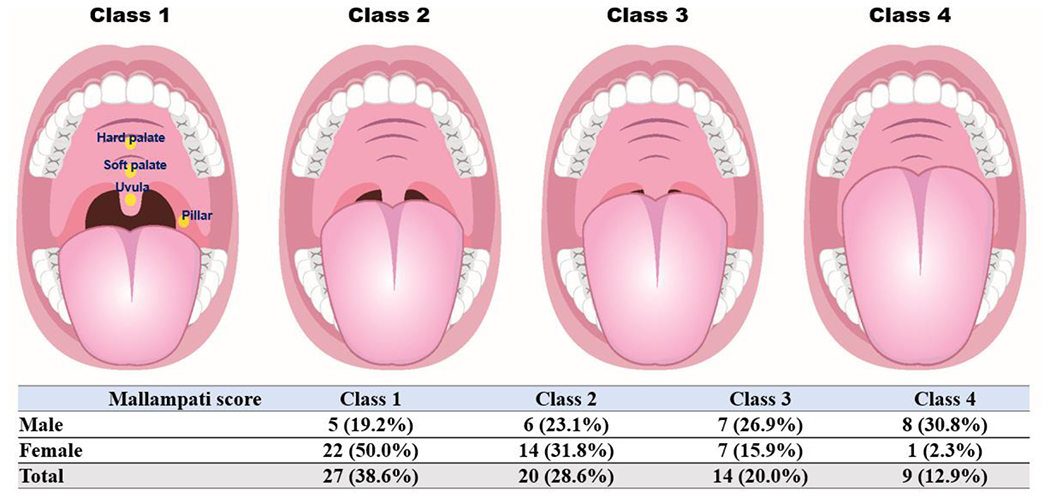
Distribution of Mallampati score according to sex in TMD patients. Class 1: Faucial/tonsillar pillars, uvula, and soft palate are all visible. Class 2: Partial visibility of the faucial/tonsillar pillars, uvula, and soft palate. Class 3: Base of the uvula, soft palate, and hard palate are visible. Class 4: Only the hard palate is visible.

The Mallampati score (Mallampati classification) is a simple test, possibly a sensitive predictor of OSA [22]. The Mallampati score is based on inspection of the upper airway. The patient was instructed to open the mouth, while protruding the tongue, to their best possible limit. Scoring depends on the visibility of anatomical structures in the oropharynx, and Mallampati scores are distributed as follows (Figure 1): 1, the soft palate and entire uvula are visible; 2, the soft palate, hard palate, and upper portion of the uvula are visible; 3, the soft palate; hard palate; and base of the uvula are visible; 4, only the hard palate is visible. For every 1-point increase in the Mallampati score, the odds of having OSA, increased by more than 1.5 folds [23].

### Portable PSG index for snoring and OSA

**Figure 2:**
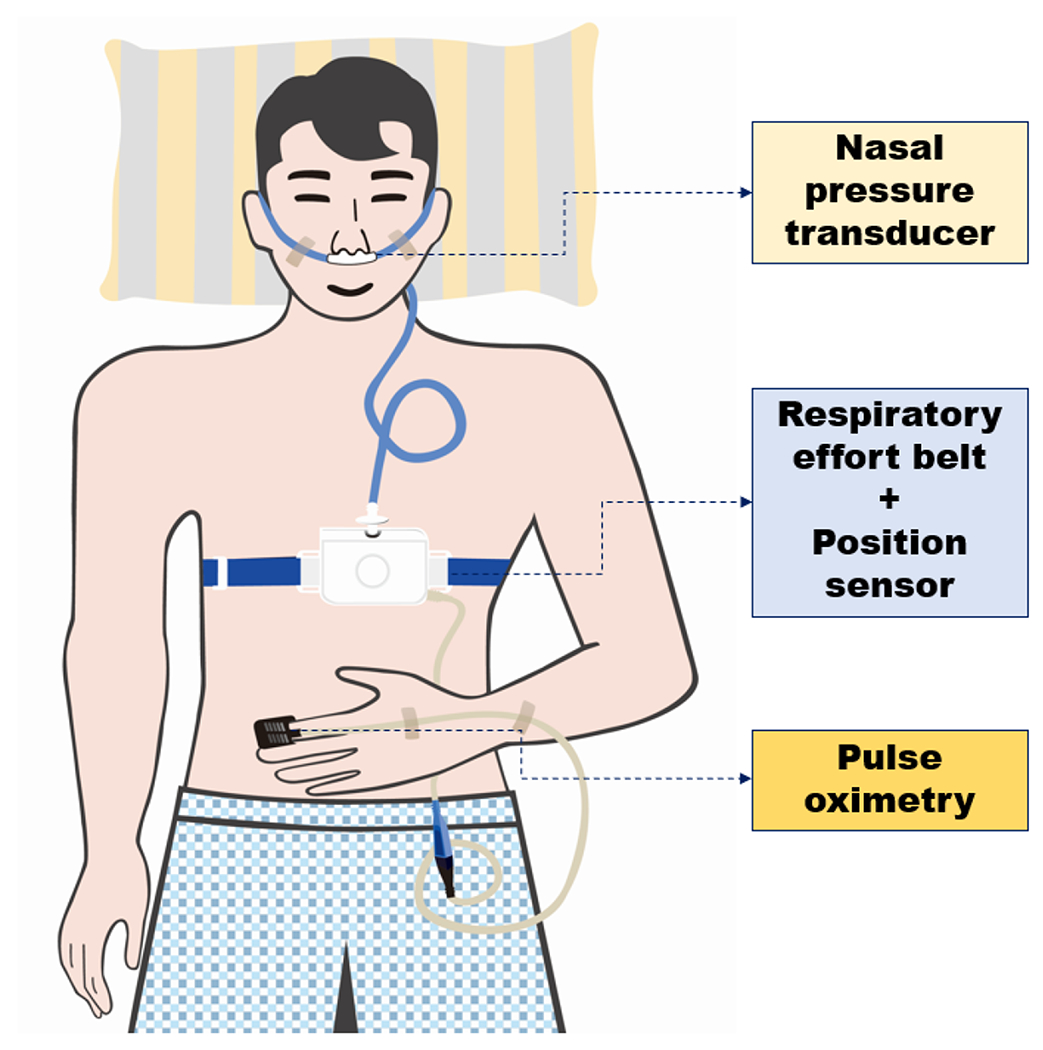
Schematic diagram of a study participant equipped with portable polysomnography

We identified and diagnosed OSA using Alice OneNight (Philips, Amsterdam, The Netherlands). Alice OneNight home sleep testing is a portable level 3 PSG device used to test sleep apnea at home. It includes oxygen saturation (saturation of percutaneous oxygen (SpO_2_), finger probe, and oximetry board Nonin), pulse rate (from oximeter probe), airflow (pressure-based airflow with detection of snoring through a nasal cannula and thermistor), thoracic and abdominal effort (inductance plethysmography), and body position (Figure 2). The patient installed and operated this device on their body for more than 1 day, and the operator selected representative data for 1 day without data loss and used them for analysis.

The presence of snoring, total time spent snoring, percentage of snoring time (ratio of total time spent snoring to total sleep time), total number of snoring episodes, and average snoring episode time were obtained and analyzed. The respiratory event index (REI) was used to predict OSA. REI represents the number of apneas and hypopneas detected by the portable monitoring device per hour of elapsed recording time. The central apnea index (CAI) and obstructive apnea index (OAI) were added, and each index was used to calculate the number of events per hour. The number of events per hour of mixed sleep apnea was also determined. Mixed sleep apnea is a combination of obstructive and central sleep apnea [24]. Apnea was defined as a 90% reduction in airflow for at least 10 s, and hypopnea was defined as a ≥30% reduction in airflow for at least 10 s, associated with a ≥3% reduction in oxygen saturation. OSA was defined as an REI ≥5/h, and while the normal REI was <5/ h) [25]. Sleep studies were considered acceptable for data analysis if none of the following criteria were met: (1) portable monitoring device with elapsed time <2 h or (2) poor-quality PSG recording (defined as a substantial portion of the PSG not interpretable for the scoring of sleep and respiratory events).

### Data analysis

Descriptive statistics are presented as percentages, means, and standard deviations (SDs) for continuous variables. The student’s t-test for non-normally distributed variables was used to compare the male and female TMD groups. Differences in the means of continuous variables between the independent groups were examined using the student’s t-test. The chi-square test with Bonferroni correction was used to determine the equality of the proportions. The Spearman’s correlation test was used to analyze bivariate correlations between the categorical and continuous variables. The Spearman’s correlation coefficient was expressed as Spearman’s rho (r), with an absolute value closer to 1, indicating a stronger correlation. The performance of the classification model at the defined threshold was demonstrated by plotting a receiver operating characteristic (ROC) curve, and the area under the ROC curve (AUC) value was calculated for each model. AUC values were interpreted as AUC=0.5 (no discrimination), 0.6≥ AUC >0.5 (poor discrimination), 0.7≥ AUC >0.6 (acceptable discrimination), 0.8≥ AUC >0.7 (excellent discrimination), and AUC >0.9 (outstanding discrimination) [26]. Kappa statistics were used to measure the degree of agreement (kappa coefficient) between two examiners who evaluated and rated the same subjects. Multiple logistic regression analysis was performed with snoring as a dependent variable and demographics, clinical characteristics of TMD, and other PSG parameters as independent variables. We also investigated whether there were sex-based differences in the OSA-related factors, examined using portable PSG. Odds ratios (ORs) with 95% confidence intervals (CIs) and p-values were calculated. Statistical significance was set at a two-tailed p-value of <0.05. Data were analyzed using IBM SPSS Statistics for Windows (version 20.0; IBM Corp., Armonk, NY, USA).

## Results

### Demographics and clinical characteristics of TMD patients

Demographic and clinical factors are shown in table 1. In this study, 44 female (62.9%, mean age: 46.61±17.73 years) and 26 male (37.1%, mean age: 46.81±19.27 years) TMD patients were enrolled, and there was no significant difference in age and sex. The male to female ratio was 1:1.69. BMI (male vs. female, 24.94±1.78 vs. 56.30±4.25, p<0.001), ratio of overweight (57.7% vs. 11.4%, p<0.001), and Mallampati score (2.69±1.12 vs. 1.70±0.82, p<0.001) differed according to sex, with the values being significantly higher in male than in female patients.

### Distribution of contributing factors for TMD

Among the representative contributing factors of TMD, only the presence of bruxism differed between sexes. Bruxism was found in 3.8% of male and 29.5% of female patients with TMD (p=0.012). The most common contributing factors for TMD in both male and female patients were headache (male, 53.8%; female, 54.5%), followed by psychological distress (male, 50.5%; female, 52.3%), and sleep problems (male, 34.6%; female, 36.4%) (Table 2).

### OSA diagnosis with portable PSG

Considering SpO_2_, the duration of SpO_2_ <90% was significantly higher in female than in male TMD patients (0.15±0.33 min vs. 0.53±1.12 min, p=0.043). In addition, the mixed sleep apnea index was significantly higher in female than in male TMD patients (0.81±0.80 vs. 0.44±0.54, p<0.022). There was no difference between the REI (8.84±10.41 vs. 9.21±8.39, p>0.05) and OSA presence rate based on REI ≥5 (54.5 % vs. 42.3%, p>0.05) in female and male TMD patients (Table 3).

### Snoring investigated by portable PSG

Total sleep time and that spent snoring did not differ significantly between male and female patients (295.57±188.42 min vs. 255.03±184.77 min, p=0.382 and 5.18±5.43 min vs. 3.61±3.89 min, p=0.205, respectively). In addition, the total number of snoring episodes and average snoring episode time did not differ significantly between male and female patients (16.98±41.88 vs. 16.85±34.27, p=0.806 and 23.71±14.22 s vs. 19.43±13.27 s, p=0.214, respectively). The presence of snoring was observed in 15.4% of male patients and 13.6% of female patients (p=0.398). Interestingly, the prevalence of snoring was lower than that of OSA in both sexes (Table 4).

### Multiple logistic regression analysis with clinical factors for predicting snoring

Among several factors, including age, sex, and contributing factors for TMD, female sex and macrotrauma history significantly predicted the presence of snoring. When compared to male patients, female patients showed a 0.146-fold decrease in the incidence of snoring (OR=0.146, p=0.022). In addition, macrotrauma history was found to increase the prediction of snoring by 6.165 times (OR=6.165, p=0.034). The presence of bruxism, sleep problems, headache, psychological distress, and tinnitus, along with age and VAS score, were not significant predictors of snoring (Table 5).

### Factors correlating with the presence of snoring in TMD patients

The presence of snoring showed a positive correlation with the Mallampati score in both male (r=0.818, p=0.001) and female (r=0.501, p=0.001) patients, and the degree of correlation expressed by Spearman’s r was greater in male patients. In addition to the Mallampati score, significant positive correlations were observed between OAI (r=0.788, p=0.001), REI (r=0.750, p=0.001), and snoring (Table 6), exclusively in the male patients with TMD.

The results were obtained from Spearman’s correlation analysis. Spearman’s r indicates the correlation between two factors (the range of Spearman’s r: −1 to 1). The larger the absolute value, the stronger the correlation. In this table, red indicates positive correlation and green indicates negative correlation. A p-value <0.05 was considered significant. **: p-value<0.01.

### ROC analysis predicting snoring in TMD patients

**Figure 3:**
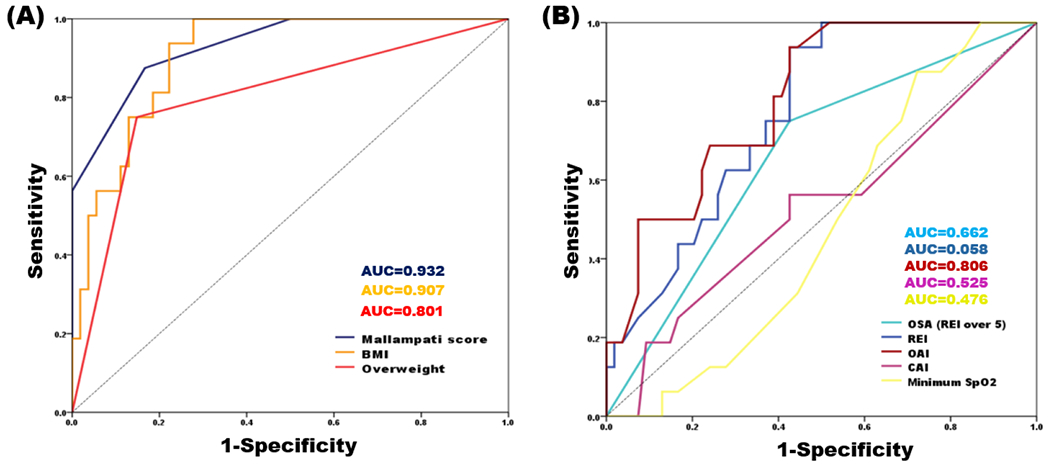
ROC analysis of significant predictors of snoring. (A) Factors related to Mallampati score, (B) factors related to OSA ROC, receiver operating characteristic; OSA, obstructive sleep apnea; BMI, body mass index; AUC, area under ROC curve; REI, respiratory event index; OAI, obstructive apnea index; CAI, central apnea index; SpO_2_, saturation of percutaneous oxygen.

For the total sample, the significance of AUC values was investigated using the ROC analysis (Table 7). Mallampati score (AUC=0.932, p<0.001) and BMI (AUC=0.907, p<0.001) strongly predicted the presence of snoring at an outstanding discrimination level (AUC>0.9) in patients with TMD. Overweight (BMI ≥25) (AUC=0.801, p<0.001), OAI (AUC=0.806, p<0.001), and REI (AUC=0.768, p=0.01) were also significant predictors with excellent discrimination levels for the presence of snoring (Figure 3). Therefore, snoring was correlated with Mallampati score, BMI, and risk of OSA.

## Discussion

In the present study, for the first time, clinical characteristics, snoring, and OSA were investigated in patients with TMD using portable PSG, and sex-based differences were comprehensively investigated in terms of their mean values, distribution, and relationships. While Mallampati score, BMI, and overweight ratio were significantly higher in male than in female TMD patients, OSA was more common than snoring in TMD patients; 50.0% of TMD patients had OSA and 15.7% had snoring, but no sex-differences were found in the prevalence of OSA and snoring. In both sexes, Mallampati score was significantly positively correlated with snoring; it was the strongest predictor of snoring, and the AUC value of 0.932 was indicative of its outstanding discrimination value. In addition, there were significant positive correlations between OAI, REI, and presence of snoring in male patients with TMD, while no correlation was observed among female patients. Furthermore, sleep in patients with TMD was also examined for PSG parameters with sex-based differences, and the mixed sleep apnea index was significantly higher in female than in male TMD patients. The results demonstrate that the causes of sleep problems, including snoring and OSA, and the correlation with BMI and other clinical characteristics may differ between female and male TMD patients.

In this study, Mallampati score was an important anatomical factor for predicting snoring in both male and female patients with TMD. It is a measure of the density of soft tissue in the oropharynx and usually increases with increasing obesity and BMI ^27^. A high Mallampati score is associated with sleep problems in the upper airway, including snoring and OSA ^28^. According to Nuckton et al., the Mallampati score may be useful as an independent predictor of OSA, which is consistent with the subsequent overnight PSG results ^22^. The mixed sleep apnea index was higher in female than in male TMD patients. In our results, there were significant positive correlations between OAI, REI, and presence of snoring, only in male TMD patients. Thus, Mallampati scoring could be a useful tool for the physical examination of patients prior to PSG ^22^ by predicting the presence of snoring in patients with TMD, while a high score could correlate with the presence of OSA in male patients.

The mixed sleep apnea index was higher in female than in male TMD patients. No previous studies have examined sex-based differences in mixed sleep apnea in TMD patients. Unlike OSA, central sleep apnea is defined as a lack of continuous breathing effort during airflow [29]. However, this distinction is sometimes rather difficult because there is a significant overlap in the etiology and pathophysiology of OSA and central sleep apnea [30]. Therefore, the concepts of mixed sleep apnea, complex sleep apnea, or sleep apnea spectrum have emerged. Mixed sleep apnea is a combination of both obstructive and central sleep apnea symptoms [31]. Although the pathogenesis of OSA and central sleep apnea is not fully understood, several anatomical and non-anatomical factors are considered to interact. The key factors include the interaction of upper airway obstruction, unstable central ventilation modulating factors, and an individual’s systemic conditions/characteristics [32]. Withdrawal of behavioral control over ventilation during sleep and blunt chemoresponsiveness to changes in arterial CO_2_ (PaCO_2_) and oxygen (PaO_2_), changes in lung volume, and ventilation due to sleep states (REM vs. non-REM) result in greater variability in PaCgO_2_ levels [33]. If the ventilatory response to the stimulus is exaggerated, PaCO_2_ can easily fall below the apnea threshold, causing central apnea. In this study, female sex was a predictor of a decrease in the incidence of snoring. The prevalence and characteristics of sleep apnea in female patients vary throughout their life span as they go through different life stages: puberty, reproductive years, pregnancy, and postmenopausal state [34]. Additionally, male and female patients have different pathophysiological factors for sleep apnea, such as upper airway anatomy, chemoreflexes, sex hormones, and the ability to recognize sleep disorders. Further studies on snoring and sleep apnea, considering these parameters in patients with TMD, are needed.

Based on the results of portable PSG, snoring was strongly correlated with OSA and REI scores in male patients with TMD. In addition, AUC values suggest that OAI and REI were major predictors of snoring. Despite not affecting sleep architecture, snoring adversely affects sleep efficiency [35]. Although snoring is considered a representative symptom of OSA, there has been no evaluation of snoring in patients with TMD. Decreased sleep efficiency may interfere with patients’ daily activities, contributing to an increase in their sensitivity to pain [36]. In this study, the clinical pain index, VAS, had no significant relationship with snoring; additional studies on TMD pain index and snoring are needed to clarify these findings. There was no significant sex-based difference in the prevalence of snoring (female, 15.4% vs. male, 13.6%, p=0.398) and OSA (female, 54.5% vs. male, 42.3%, p=0.458) in TMD patients based on portable PSG results. A bidirectional association between OSA and TMD has been proposed, which is evident from its high prevalence [37]. OSA has been reported as a common comorbidity in 37% of chronic pain patients and 28.6% of TMD patients [18]. The development and progression of snoring and OSA are closely related to obesity, measured as an increase in BMI [38]. In this study, overweight and increased BMI were observed more frequently in male than in female TMD patients. Patients with obesity (BMI ≥30) were not observed in this TMD study, and 57.7% of male and 11.4% of female patients were overweight (BMI ≥25). Considering 63% of patients to be overweight and 26% as obese [39], Korean patients with TMD have a lower percentage than American patients. The link between overweight, snoring, and OSA in TMD patients, particularly in male patients, may be due to underlying metabolic alterations and elevations in systemic inflammation.

This study had several limitations. First, patients with TMD who participated in this study willingly agreed to perform portable PSG at home, and there may be a bias depending on the patient’s propensity. Therefore, the mean age (46.69±18.18 years) of patients with TMD in this study is higher than that in our previous studies [40,41]. Second, the sample size was limited to represent the characteristics of typical TMD patients. As a result of this single-institution study, a multicenter, large-sample study was planned. Third, additional studies that consider blood tests, psychological questionnaires, and TMD pain indices are needed to support the results of this study. Additional sleep studies using laboratory-based PSG may further reinforce these findings. The significance and strength of this study is that snoring and OSA in TMD patients were objectively investigated using a portable PSG, and sex-based differences in clinical patterns and sleep-related results were uniquely identified.

## Figures and Tables

**Table 1:** Demographics and clinical characteristics of TMD patients

	Male TMD patients (n=26)	Female TMD patients (n=44)	
	mean ± SD or n (%)	mean ± SD or n (%)	p-value
Demographics			
Age (years) ^a^	46.81 ± 19.27	46.61 ± 17.73	0.966
Sex	26 (37.1%)	44 (62.9%)	
TMD index			
VAS (0-10) ^a^	5.96 ± 2.03	5.76 ± 2.02	0.114
Symptom duration (days) ^a^	593.65 ± 1054.75	829.05 ± 1613.69	0.509
BMI			
Weight (kg) ^a^	76.15 ± 4.09	56.30 ± 4.25	**<0.001** ***
Height (m) ^a^	1.75 ± 0.59	1.59 ± 0.46	**<0.001** ***
BMI (kg/m^2^) ^a^	24.94 ± 1.78	22.02 ± 2.24	**<0.001** ***
Overweight (BMI≧25) ^b^	15 (57.7%)	5 (11.4%)	**<0.001** ***
Mallampati score for the prediction of OSA			
Mallampati score (1-4) ^a^	2.69 ± 1.12	1.70 ± 0.82	**<0.001** ***

TMD: temporomandibular disorder, BMI: body mass index, VAS: visual analogue scale, OSA: obstructive sleep apnea, SD: standard deviation.

a: The results were obtained via t-test.

b: The results were obtained from chi-square test. A p-value <0.05 was considered significant.

***: p-value<0.001.

**Table 2:** Distribution of contributing factors for TMD

	Male TMD patients (n=26)	Female TMD patients (n=44)	
	mean ± SD or n (%)	mean ± SD or n (%)	p-value
*Contributing factor*			
Bruxism^a^	1 (3.8%)	13 (29.5%)	**0.012** *
Sleep problem^a^	9 (34.6%)	16 (36.4%)	1
Headache^a^	14 (53.8%)	24 (54.5%)	1
Psychological distress^a^	13 (50.0%)	23 (52.3%)	1
Tinnitus^a^	2 (7.7%)	11 (25.0%)	0.111
Macrotrauma^b^	7 (26.9%)	6 (15.9%)	0.356

TMD: temporomandibular disorder, A p-value <0.05 was considered significant.

*: p-value<0.05,

**: p-value<0.01,

***: p-value<0.001.

a: The results were obtained from chi-square test.

b: The results were obtained from Fisher’s exact test and Bonferroni correction.

c: The results were obtained via t-test.

**Table 3:** Investigation of OSA with portable polysomnography

	Male TMD patients (n=26)	Female TMD patients (n=44)	
	mean ± SD or n (%)	mean ± SD or n (%)	p-value
**Saturation of oxygen**			
Lowest SpO_2_ (%)^a^	81.50 ± 24.21	94.36 ± 1.98	0.634
Mean SpO_2_ (%)^a^	94.65 ± 0.89	94.65 ± 0.89	0.404
**SpO_2_ < 90% (min)** ^a^	0.15 ± 0.33	0.53 ± 1.12	**0.043** *
**Sleep apnea index**			
**Mixed sleep apnea (events/hour)** ^a^	0.44 ± 0.54	0.81 ± 0.80	**0.022** *
OAI (events/hour)^a^	5.31 ± 6.76	4.83 ± 5.16	0.755
CAI (events/hour)^a^	0.59 ± 0.60	1.41 ± 4.06	0.195
REI (events/hour)^a^	8.84 ± 10.41	9.21 ± 8.39	0.876
**Prediction of OSA**			
Normal (REI < 5)^b^	15 (57.7%)	20 (45.5%)	0.458
OSA (REI ≧ 5)^b^	11 (42.3%)	24 (54.5%)	

TMD: temporomandibular disorder, PSG: polysomnography, SpO_2_: saturation of percutaneous oxygen, OSA: obstructive sleep apnea, OAI, obstructive apnea index, CAI, central apnea index, REI: respiratory event index.

a : The results were obtained via t-test.

b : The results were obtained from chi-square test. A p-value <0.05 was considered significant.

* : p-value<0.05.

**Table 4:** Investigation of snoring by portable polysomnography

	Male TMD patients (n=26)	Female TMD patients (n=44)	
	mean ± SD or n (%)	mean ± SD or n (%)	p-value
Portable PSG index			
Total sleep time (min)^a^	295.57 ± 188.42	255.03 ± 184.77	0.382
Total spent snoring (min)^a^	5.18 ± 5.43	3.61 ± 3.89	0.205
Percentage of snoring time (%)^a^	1.74 ± 2.88	1.42 ± 2.11	0.398
Total number of snoring episodes^a^	16.98 ± 41.88	16.85 ± 34.27	0.806
Average snoring episode time (sec)^a^	23.71 ± 14.22	19.43 ± 13.27	0.214
The presence of snoring^b^	5 (15.4%)	0.398	

TMD: temporomandibular disorder, PSG: polysomnography, SpO_2_: saturation of percutaneous oxygen.

a: The results were obtained via t-test.

b: The results were obtained from chi-square test. A p-value <0.05 was considered significant.

**Table 5:** Multiple logistic regression analysis for predicting snoring in TMD patients

	Predicting snoring in TMD patients					
	OR	95% CI Lower	95% CI Upper	B	SE	p-value
Age [ref.=under average value]	1.22	0.147	10.123	0.199	1.08	0.854
Female [ref.=male]	0.146	0.028	0.757	−1.926	0.84	**0.022** *
VAS [ref.=under average value]	0.177	0.032	0.987	−1.734	0.888	0.051
Bruxism [ref.=none]	1.841	0.457	7.412	0.61	0.711	0.39
Sleep problem [ref.=none]	0.56	0.13	2.404	−0.58	0.744	0.435
Headache [ref.=none]	0.169	0.014	2.097	−1.781	1.286	0.166
Psychological distress [ref.=none]	0.657	0.1	4.332	−0.421	0.963	0.662
Tinnitus [ref.=none]	0.297	0.07	1.256	−1.213	0.735	0.099
Macrotrauma [ref.=none]	6.165	1.143	33.27	1.819	0.86	**0.034** *
Constant	1.442	-	-	0.366	0.911	0.688

TMD: temporomandibular disorder, VAS: visual analogue scale, OR: odds ratio, CI: confidence interval, SE: Standard error, B: logistic regression coefficient

a: The results were obtained via t-test.

b: The results were obtained from chi-square test. A p-value <0.05 was considered significant.

*: p-value<0.05

**Table 6:** Factors correlated with the presence of snoring in TMD patients

Parameters		Male TMD patients (n=26)	Female TMD patients (n=44)
		The presence of snoring	The presence of snoring
Mallampati score	Spearman’s r	**0.818**	**0.501**
	p-value	0.001**	0.001**
OAI	Spearman’s r	0.788**	0.195
	p-value	0.001**	0.204
CAI	Spearman’s r	0.055	0.057
	p-value	0.789	0.711
REI	Spearman’s r	**0.75**	0.155
	p-value	0.001**	0.315

TMD: temporomandibular disorder, OSA: obstructive sleep apnea, OAI, obstructive apnea index, CAI, central apnea index, REI: respiratory event index.

**Table 7:** ROC analysis predicting snoring in TMD patients

	AUC	SD	p-value	95% CI	
				Lower	Upper
Clinical factors					
Mallampati score	**0.932**	0.033	**<0.001** ***	0.867	0.997
BMI	**0.907**	0.035	**<0.001** ***	0.84	0.975
Overweight (BMI≧25)	**0.801**	0.069	**<0.001** ***	0.665	0.937
Polysomnography factors					
Minimum SpO_2_	0.476	0.071	0.774	0.337	0.616
OAI	**0.806**	0.055	**<0.001** ***	0.698	0.914
CAI	0.525	0.086	0.758	0.357	0.694
REI	**0.768**	0.058	**0.001** **	0.655	0.881
Snoring (REI > 5)	0.662	0.076	0.05	0.514	0.81

TMD: temporomandibular disorder, ROC: Receiver operating characteristic, AUC: area under the ROC curve, SD: standard deviation, CI: confidence interval, BMI: body mass index, OAI, obstructive apnea index, CAI, central apnea index, REI: respiratory event index, SpO_2_: saturation of percutaneous oxygen.

The results were obtained by the ROC curve analysis. A p-value <0.05 was considered significant.

**: p-value<0.01

***: p-value<0.001.

## Data Availability

Owing to the sensitivity of patient data, the KHU-IRB will discuss any requests before disclosure.
